# Attachment and psychopathology in children and adolescents: a cross-sectional study of children with type 1 diabetes and their healthy peers

**DOI:** 10.3389/fpsyg.2026.1630917

**Published:** 2026-02-19

**Authors:** Anja Turin Drouet, Maja Drobnič Radobuljac, Nataša Bratina, Sašo Karakatič, Tadej Battelino, Klemen Dovč, Simona Klemenčič

**Affiliations:** 1 Department for Child Psychiatry, University Children’s Hospital Ljubljana, University Medical Centre Ljubljana, Ljubljana, Slovenia; 2 Faculty of Medicine, University of Ljubljana, Ljubljana, Slovenia; 3 Centre for Mental Health, University Psychiatric Clinic Ljubljana, Ljubljana, Slovenia; 4 Department of Pediatric Endocrinology, Diabetes and Metabolic Diseases, University Children’s Hospital Ljubljana, University Medical Centre Ljubljana, Ljubljana, Slovenia; 5 Institute of Informatics, Faculty of Electrical Engineering and Computer Science, University of Maribor, Maribor, Slovenia

**Keywords:** attachment security, child/adolescent, psychopathology, stressful life events, type 1 diabetes

## Abstract

**Introduction:**

Secure attachment develops in early relationships between infants and their caregivers, providing a foundation for emotional security and mental health across the lifespan. In contrast, insecure attachment is associated with maladaptive stress response and an increased risk of both internalizing and externalizing mental health problems. This study examined the association between attachment (in)security and psychopathology in children with type 1 diabetes (T1D) compared with healthy peers. We also considered caregivers’ attachment security and traumatic life events to better understand interacting biopsychosocial factors in children living with a chronic illness.

**Methods:**

A group of children with T1D (*N* = 101) and a group of healthy control children (*N* = 106) aged 8–15 years and one of their parents were included in the study. A Child Attachment Interview (CAI) was conducted with the children and a Relationship Structures Questionnaire (ECR-RS) with the parents to assess their attachment security. Stressful life events in the children’s lives were recorded using a questionnaire on the traumatic events (LITE). The children’s psychopathology was assessed using the Child Behavior Checklist (CBCL) and the Youth Self-Report (YSR). Descriptive analyses and linear regression models were used to analyze the data.

**Results:**

Fifty-nine percent of children had secure attachment, 39% were dismissing, and 2% preoccupied; 16% were classified as disorganized. No significant difference in psychopathology was found between children with T1D and their healthy peers. However, insecure/disorganized attachment was associated with higher psychopathology scores. Simple regression showed positive associations between psychopathology and child attachment insecurity, maternal attachment anxiety, and traumatic life events. A more complex model revealed that male sex, the presence of T1D, maternal attachment anxiety in older children, and the interaction between maternal attachment anxiety and traumatic life events were significantly associated with higher levels of psychopathology.

**Conclusion:**

Children with insecure, particularly disorganized, attachment showed higher levels of psychopathology. However, when interaction effects were considered, other factors—such as sex, parental attachment, traumatic life events, and chronic illness—emerged as stronger predictors. These findings highlight the importance of attachment- and trauma-informed care that addresses multiple risk factors to support child and family mental health.

**Clinical trial registration:**

ClinicalTrials.gov, NCT02575001.

## 1 Introduction

Interaction with an empathetic and attentive caregiver in the first years of life enables a child to develop a secure attachment, defined as an organized attachment representation characterized by expectations of caregiver availability, responsiveness, and emotional support, and reflected in positive mental representations of self and others. In contrast, insecure attachment reflects an attachment representation shaped by inconsistent, unavailable, or rejecting caregiving, resulting in negative expectations regarding the self and attachment figures (Bretherton and Munholland, 2008). Children with insecure attachment are more likely to struggle with adapting to environmental challenges and are at increased risk for internalizing and externalizing psychopathology (Brumariu and Kerns, 2010; Fearon et al., 2010). In stressful situations, insecurely attached individuals tend to rely on secondary attachment strategies: hyperactivation, involving intense pursuit of closeness coupled with mistrust of the attachment figure, often leading to heightened distress and anger; or deactivation, characterized by avoidance of closeness, denial of support needs, and emotional withdrawal (Fearon et al., 2010). These less adaptive strategies reflect impaired self-regulation, which constitutes a transdiagnostic vulnerability for psychopathology (Groh and Narayan, 2019; Messina et al., 2023).

According to standardized measures, insecure attachment is subclassified into three categories reflecting different strategies for regulating attachment needs: avoidant (dismissing), anxious (preoccupied), and disorganized (Shmueli-Goetz et al., 2008). Disorganized attachment is characterized by a lack of a coherent strategy for seeking comfort and regulating distress in the presence of an attachment figure. It is frequently observed in children exposed to maltreatment, trauma, or frightening or frightened caregiving and has been consistently associated with an increased risk for later psychopathology (Green and Goldwyn, 2002).

Type 1 diabetes (T1D) is one of the most common chronic diseases in childhood, and its incidence continues to rise worldwide (Atkinson et al., 2014; Patterson et al., 2019). Managing T1D requires strict adherence to treatment, lifestyle adjustments, and continuous monitoring to prevent acute and chronic complications (Turin and Drobnič, 2021). These management demands represent a significant source of psychological stress for children, as well as their caregivers, whose own attachment representations may influence how effectively they regulate stress and provide support (Turin et al., 2021; Klemencic et al., 2023). Mothers of children with T1D were frequently reporting the presence of post-traumatic stress symptoms that can persist for years (Rechenberg et al., 2017). Previous studies have yielded inconsistent findings regarding the prevalence of mental health issues in children and adolescents with T1D compared to healthy controls: some large surveys report higher rates of depression, anxiety, and eating disorders, whereas others report similar or lower rates, despite consistent evidence that psychopathology undermines effective diabetes management (Butwicka et al., 2015; Sivertsen et al., 2014). Because insecure attachment, particularly disorganized attachment, has been associated with higher rates of psychopathology (Green and Goldwyn, 2002), this raises the question of whether similar associations may be observed in children with T1D, particularly in the context of chronic illness–related stress and caregiving quality shaped by caregivers’ stress regulation and attachment representations. A few previous studies have examined the relationship between child attachment and T1D outcomes. Rosenberg and Shields, in a pilot study of 31 families of adolescents with T1D, found a positive correlation between maternal reports of the child’s secure attachment and metabolic control (HbA1c) (Rosenberg and Shields, 2009). Costa-Cordella et al. (2020) included 77 mother–child pairs and found that children’s secure attachment correlated with better glycaemic control, but only in boys, suggesting potential sex differences in the impact of attachment on diabetes management. In our previous work, we observed that suboptimal glycaemic control occurred in boys who were insecurely attached and in securely attached girls, possibly reflecting differences in diabetes care responsibilities within trusting parent–child relationships.

Drawing on these assumptions and our prior theoretical and empirical work, we hypothesized that insecure attachment, in combination with stress imposed by T1D on both children and their caregivers, may increase the risk of psychopathology (Turin and Drobnič, 2021; Turin et al., 2021; Klemencic et al., 2023). Accordingly, the present study aimed to examine the relationship between attachment quality and psychopathology in children with T1D compared to healthy peers. Attachment was conceptualized using two distinct constructs: child attachment, reflecting the child’s attachment representations toward the caregiver, and caregiver attachment, representing caregivers’ own attachment representations and relational patterns shaped by experiences with their own caregivers or other significant attachment figures. Within a biopsychosocial framework, exposure to traumatic life events was included as an additional factor relevant to child psychopathology (Turin and Drobnič, 2021; Turin et al., 2021; Klemencic et al., 2023).

## 2 Materials and methods

### 2.1 Participants and procedures

We conducted a case–control observational study that included all eligible children from the Slovenian National Registry of Childhood T1D and their parents or caregivers, as well as a healthy control group of peers. The main inclusion criteria for the cases were an age between 8 and 15 years and a duration of T1D of more than 1 year. The exclusion criteria were intellectual disability and active psychosis. We invited the study group by mail, followed by a telephone invitation by their diabetologist before attending the regular three-monthly check-up. The control group consisted of children of the same age group who did not have T1D. They attended five randomly selected elementary school from across the country. The nature of the study was explained to the parents and children by one of the researchers or school counsellors. Parents were also given a written information sheet explaining the study.

Inclusion in the survey began in July 2015 and ended in December 2019. The invitation was sent to 124 families of children with T1D, 101 chose to participate. 380 children and one of their parents or caregivers were invited to the control group, 115 chose to participate, 9 of whom withdrew during the study. Participation was voluntary and anonymous, and all participants and their parents signed a consent form prior to participation. The recording of the interviews and the assessments were conducted at the university pediatric clinic (the cases) or at school before the start of classes (control group).

### 2.2 Measures

#### 2.2.1 Attachment representations

Child attachment representations were measured using a semi-structured interview, whereas caregivers’ attachment representations—reflecting their own attachment-related experiences with their caregivers or other significant attachment figures—were measured using a questionnaire.

#### 2.2.2 Child attachment interview (CAI)

The CAI is a narrative-based psychological instrument to assess children’s and adolescents’ internal working models of attachment (Shmueli-Goetz et al., 2008). Children are asked to describe and reflect on their current attachment relationships. The interview is intended for 8- to 15-year-olds. It is assessed by analyzing interview transcripts and video recordings. Children or adolescents are then classified in a two-way classification as securely or insecurely attached, in a three-way classification as secure, preoccupied, or dismissing, and in a four-way classification as secure, preoccupied, dismissing, or disorganized (Shmueli-Goetz et al., 2008). Due to the small sample size, only two-way classifications were used in all analyses; similar decisions have been made by other researchers (Costa-Cordella et al., 2020; Bizzi et al., 2021). In addition, disorganization was used as an independent variable in our models. The protocols were scored by three independent accredited coders, who showed high inter-rater reliability as previously reported (Turin et al., 2021).

#### 2.2.3 Relationship structures questionnaire (ECR-RS)

This questionnaire contains nine questions to assess attachment to each of the four attachment figures: both parents, partner, best friend. The general attachment is calculated as the average of the results for all figures (Rocha et al., 2017). Within each relationship domain, the questionnaire assesses two dimensions: attachment-related anxiety (how worried the person is that the attachment figure might reject them) and attachment-related avoidance (what kind of strategies the person uses to regulate their attachment behavior in the relationship context, from comfort with using others as a secure base and “safe haven” to discomfort with closeness and dependence) (Fraley and Hudson, 2017). The securely attached person scores low on both dimensions. The reliability for the dimensions is high to excellent (Cronbach’s alpha above 0.7 for various attachment figures and areas) (Rocha et al., 2017). The questionnaire in our research was completed by one of the parents.

#### 2.2.4 Questionnaire of general sociodemographic data

The questionnaire contained 27–30 questions (the latter for parents of children with T1D, including questions on T1D management) covering general demographic and family characteristics. It was administered to parents or caregivers and included items on sex, age of the child, parents’ age, education and employment status, size of the town of residence, presence of chronic or mental illness in parents, death in the family, and related factors. The questionnaire was adapted from a risk-factor questionnaire that has been used in Slovenia since 1996 (Drobnič Radobuljac et al., 2009). A shortened version of the questionnaire was used, with additional questions on T1D management (duration of T1D, most recent glycated haemoglobin (HbA1c) level, use of an insulin pump), current health status, and development during the first year after birth. The questionnaire in our research was completed by one of the parents.

#### 2.2.5 Child behavior checklist (CBCL)

The CBCL is a validated instrument for the assessment of psychopathology in children and adolescents (Copyright 2007, T.M. Achenbach ASEBA, University of Vermont, 1 South Prospect St. Burlington, VT 05401-3456)^1^. The questionnaire contains 170 questions and is administered in three slightly adapted formats to parents (CBCL/6-18), adolescents aged 11 and older (YSR) and teachers (TRF). The questionnaire has been validated and officially translated into Slovenian. The questions are grouped to assess the presence of various symptoms, which are presented in scales. The eight Achenbach scales that are most used are: Anxiety/Depression, Withdrawal/Depression, Somatic Complaints, Difficulties in Social Relationships, Thought Disorders, Attention Disorders, Rule Violations, and Aggressive Behavior, which are further divided into groups of internalizing, externalizing, and other symptoms (Achenbach et al., 2008). CBCL and YSR scales show strong psychometric properties, with internal consistency coefficients (Cronbach’s *α*) typically ranging from 0.71 to 0.97 across syndrome and broadband scales, and test–retest correlations from 0.74 to 0.97, indicating high reliability and stability over time (Achenbach and Rescorla, 2001). The CBCL questionnaire in our research was completed by one of the parents and the YSR by children aged 11 and older.

#### 2.2.6 Lifetime incidence of traumatic events (LITE)

It allows assessment of the number of traumatic life events (e.g., car accident, death in the family, violence, sexual abuse, robbery) a person’s life and their emotional involvement at the time of the event and at the time of completing the questionnaire. The instrument is available in two formats: for children and for parents (LITE-Y, LITE-P). It’s test–retest reliability in the Swedish population for the total scale was found to be 0.76, and kappa per item ranged between 0.33 and 0.86 (Nilsson et al., 2010). The questionnaire was officially translated for the purposes of the survey and validated in the Slovenian population, where retest reliability for individual scales was *r* = 0.469–0.639 (*ρ* = 0.443–0.636), but higher for individual items (*κ* = 0.263–0.821) (Uršič et al., 2021). The questionnaire in our research was completed by one of the parents.

### 2.3 Statistical analysis

We performed descriptive comparisons between cases and controls in terms of age, sex, attachment classification and psychopathology, as well as comparisons between the securely and insecurely attached groups and the female and male sex in terms of psychopathology. *T*-tests for independent samples, Mann–Whitney *U*-tests, Pearson chi-square tests and Fischer exact tests were used in the IBM SPSS statistical package for continuous or categorical variables. The linear regression model in SPSSS and the statistical program R were then used to predict psychopathology (CBCL). The independent variables used in the models were: T1D, a dichotomous attachment classification (secure or insecure), presence of disorganized attachment, attachment-related anxiety of the parents (ECR-RS anxiety) and stressful life events of the child (LITE-P). In a second phase, sex, age, and interactions between the variables were added as described above.

Before multivariate modeling, we assessed bivariate associations between psychopathology (CBCL) and the following candidate predictors: T1D, attachment classification (secure vs. insecure), disorganized attachment, parental attachment-related anxiety (ECR-RS anxiety), child stressful life events (LITE-P), age, and sex. Multivariate linear regression models were used to assess the correlations between the independent variables and psychopathology. The independent variables were selected according to two criteria: (i) statistically significant relationship with the dependent variable and (ii) selection of variables based on theoretical background (enter method)—T1D, child attachment classification (secure/insecure) and disorganized attachment, parental attachment-related anxiety (ECR-RS anxiety), and child stressful life events (LITE-P). Second, the stepwise procedure was used to better understand the relationships between additional predictors influencing the presence of psychopathology. In the stepwise procedure, our model included all main effect terms and all combinations between pairwise variables as interaction terms. The stepwise procedure was described in our previous reports (Turin et al., 2021). Age was entered as a continuous variable. For significant interactions involving continuous moderators, we used simple slope analyses to estimate conditional effects at the mean of the moderator and at ±1 SD.

## 3 Results

The questionnaires intended for parents were, in most cases, completed by mothers (17% by fathers and one by a grandmother).

### 3.1 Demographic data

207 children aged 8–15 years (mean 11.74 ± 2.08) took part in the study. 101 children had T1D (49.5% female), and 106 children (55.7% female) were healthy controls. There were no statistically significant differences between the groups of children with T1D and their healthy peers in terms of demographic data and attachment security, the only difference was the educational level of both parents, which was statistically significantly higher in the control group (*p* < 0.01) (Table 1) (Turin et al., 2021). This comparison has been previously reported (Turin et al., 2021). The comparison of the two groups also showed no statistically significant differences in terms of psychopathology in all categories assessed by CBCL or YSR (*p* > 0.05) (Table 2).

**Table 1:** Comparison of general demographic data between groups of children with T1D and healthy controls. Adapted with permission from Turin et al. (2021), licensed under CC BY.

Characteristic	Children with T1D	Controls	*p*
	*N* = 101	*N* = 106	
Age in years	11.8 ± 2.1	11.6 ± 2.1	0.656
Female gender	50 (49.5)	59 (55.7)	0.375
Duration of T1D in years	5.2 ± 3.4	/	/
Age of mother in years	41.1 ± 5.1	42.3 ± 4.4	0.112
Age of father in years	43.9 ± 6.3	45.1 ± 5.7	0.053
Divorced family/living with one parent/living outside the family	*N* = 96 19 (19.8)	*N* = 102 15 (14.7)	0.251
Mother education level Finished secondary school Finished university	*N* = 93 48 (51.6) 22 (23.7)	*N* = 101 18 (17.8) 70 (69.3)	**0.0001**
Father education level Finished secondary school Finished university	*N* = 93 57 (61.3) 15 (16.1)	*N* = 98 32 (32.7) 45 (45.9)	**0.0001**
Mother employed	*N* = 94 80 (85.1)	*N* = 99 93 (93.9)	0.068
Father employed	*N* = 93 81 (87.1)	*N* = 98 92 (93.9)	0.600
CAI CAI Secure	*N* = 101 65 (64.4)	*N* = 106 56 (52.8)	0.093
CAI Insecure Dismissing Preoccupied* Disorganized	36 (35.6) 25 (24.7) 0* 11 (10.9)	50 (47.2) 29 (27.4) 0* 21 (19.8)	0.093 0.670 0.076
LITE–S	*N* = 95 2.7 ± 1.8	*N* = 101 2.9 ± 1.8	0.375
ECR-RS ECR-RS avoidance ECR-RS anxiety	*N* = 93 2.6 ± 0.9 1.8 ± 0.9	*N* = 101 2.5 ± 0.9 1.5 ± 0.7	0.841 0.076

Data are *n* (%) or mean ± SD unless stated otherwise. CAI—child attachment interview, LITE: lifetime incidence of traumatic events (S = student version), ECR-RS—relationship structures questionnaire; ECR-RS avoidance–common score for avoidance in mothers, ECR-RS-anxiety = ECR-RS relationship structures questionnaire–common score for anxiety in mothers, *One (1%) of the cases and three (2.8%) of controls were classified as preoccupied in the 3-way classification but all of them were disorganized in the 4-way classification. Between-group comparisons: *T*-test, Mann–Whitney *U*-test, Pearson chi-square test and Fischer’s exact test, statistical significance *p* < 0.05 (bold).

**Table 2:** Comparison of psychopathology between groups of children with T1D and healthy controls.

CBCL scales	Children with T1D	Controls	*p*	YSR scales	Children with T1D	Controls	*p*
	*N* = 94	*N* = 100			*N* = 65	*N* = 63	
CBCL anxious/depressed	2.80 ± 2.89 (2.00)	2.34 ± 2.07 (2.00)	0.462	YSR anxious/depressed	5.09 ± 4.46 (4.00)	5.06 ± 3.98 (4.00)	0.843
CBCL withdrawn/depressed	1.65 ± 1.86 (1.00)	1.40 ± 1.79 (1.00)	0.215	YSR withdrawn/depressed	3.22 ± 2.57 (3.00)	3.11 ± 2.90 (3.00)	0.587
CBCL somatic complaints	1.24 ± 1.56 (1.00)	1.46 ± 1.84 (1.00)	0.306	YSR somatic complaints	3.23 ± 2.84 (3.00)	2.46 ± 2.19 (2.00)	0.123
CBCL social problems	1.81 ± 2.29 (1.00)	1.13 ± 1.53 (1.00)	0.050	YSR social problems	2.94 ± 2.60 (2.00)	2.68 ± 2.56 (2.00)	0.611
CBCL thought problems	1.31 ± 1.60 (1.00)	1.74 ± 1.97 (1.00)	0.171	YSR thought problems	3.38 ± 2.80 (3.00)	4.44 ± 3.50 (4.00)	0.107
CBCL attention problems	2.99 ± 3.06 (2.00)	2.84 ± 3.31 (1.00)	0.460	YSR attention problems	4.34 ± 2.80 (4.00)	5.13 ± 3.66 (4.00)	0.354
CBCL rule-breaking behavior	1.54 ± 1.98 (1.00)	1.28 ± 1.57 (1.00)	0.519	YSR rule-breaking behavior	2.66 ± 2.46 (2.00)	2.76 ± 2.10 (2.00)	0.518
CBCL aggressive behavior	3.73 ± 3.72 (2.00)	3.01 ± 3.43 (2.00)	0.132	YSR aggressive behavior	4.91 ± 3.43 (5.00)	5.54 ± 3.86 (5.00)	0.433
CBCL internalizing problems	5.67 ± 4.93 (4.00)	5.07 ± 4.19 (4.00)	0.467	YSR internalizing problems	11.54 ± 8.26 (10.00)	10.63 ± 7.46 (9.00)	0.635
CBCL externalizing problems	5.38 ± 5.34 (4.00)	4.29 ± 4.62 (3.00)	0.102	YSR externalizing problems	7.57 ± 5.24 (7.00)	8.30 ± 5.34 (7.00)	0.525
CBCL total psychopathology	18.98 ± 15.34 (14.00)	16.88 ± 13.24 (14.00)	0.397	YSR total psychopathology	32.94 ± 19.48 (31.00)	33.63 ± 20.82 (29.00)	0.934

Data are mean ± standard deviation (median). CBCL = child behavior checklist, YSR = youth self-report. Between-group comparisons: Mann–Whitney *U*-test.

Of the children who took part in the survey, 121 (59%) were securely attached. The majority of insecurely attached children were dismissing (39%) in a 3-way classification. In a 4-way classification, 16% of the children were disorganized (Table 3). Among the securely attached children, there was a higher percentage of girls (62%). The difference was statistically significant (*p* < 0.01). No additional significant differences were observed between securely and insecurely attached children with respect to general demographic characteristics (Table 4). Disorganization in attachment was statistically significantly associated with parental attachment insecurity (higher scores for attachment anxiety and avoidance, Table 4).

**Table 3:** Distribution of attachment to the mother.

Two-way classification on CAI	3-way classification on CAI	4-way classification on CAI
Secure: 121 (59%) Insecure: 84 (41%)	Secure: 121 (59%) Dismissing: 80 (39%) Preoccupied: 4 (2%)	Secure: 119 (58%) Dismissing: 54 (26%) Preoccupied: 0 (0%) Disorganized: 32 (16%)

Data are *N* (%). *N* = 205.

**Table 4:** Comparison of general demographic data between groups of securely and insecurely attached children.

Variable	CAI 2–way classification		*p*	CAI 4–way classification		*p*
	Securely attached children	Insecurely attached children		Secure/dismissing/preoccupied	Disorganized	
	*N* = 121 (59%)	*N* = 84 (41%)		*N* = 173	*N* = 32	
Female	75 (62%)	33 (39%)	**0.001**	91 (53%)	17 (53%)	0.957
	*N* = 120	*N* = 82		*N* = 171	*N* = 31	
Age in years	11.60 ± 2.12	11.88 ± 2.01	0.394	11.73 ± 2.08	11.61 ± 2.09	0.838
	*N* = 114	*N* = 77		*N* = 161	*N* = 30	
ECR-RS avoidance	2.44 ± 0.84	2.63 ± 1.03	0.266	2.44 ± 0.88	2.93 ± 1.08	**0.010**
ECR-RS anxiety	1.61 ± 0.72	1.66 ± 0.94	0.581	1.57 ± 0.77	1.96 ± 0.97	**0.021**
	*N* = 121	*N* = 84		*N* = 173	*N* = 32	
LITE-S	2.98 ± 1.73	2.90 ± 1.74	0.739	2.88 ± 1.69	3.28 ± 1.94	0.200
	*N* = 114	*N* = 80		*N* = 164	*N* = 30	
LITE-P	2.33 ± 1.74	2.41 ± 1.68	0.705	2.30 ± 1.70	2.73 ± 1.72	0.164
	*N* = 117	*N* = 80		*N* = 67	*N* = 30	
Divorced family	16 (14%)	16 (20%)	0.237	25 (37%)	7 (23%)	0.282
Mother’s education level	*N* = 115	*N* = 80		*N* = 165	*N* = 30	
*Finished secondary school*	37 (32%)	29 (36%)	0.554	57 (34%)	9 (30%)	0.628
*Finished university*	53 (46%)	40 (50%)	0.590	79 (48%)	14 (47%)	0.903
Father’s education level	*N* = 115	*N* = 75		*N* = 162	*N* = 28	
*Finished secondary school*	58 (50%)	30 (40%)	0.159	74 (46%)	14 (50%)	0.672
*Finished university*	33 (29%)	27 (36%)	0.290	52 (32%)	8 (29%)	0.711
	*N* = 115	*N* = 79		*N* = 165	*N* = 29	
Mother employed	104 (90%)	70 (89%)	0.681	147 (89%)	27 (93%)	0.744
	*N* = 114	*N* = 76		*N* = 162	*N* = 28	
Father employed	105 (92%)	67 (88%)	0.363	149 (92%)	23 (82%)	0.152

Data are mean ± SD or *N* (%); T1D: type 1 diabetes, ECR-RS: relationship structures questionnaire, LITE: lifetime incidence of traumatic events (S = student and P = parent version), CBCL: child behavior checklist, YSR: adolescent version of CBCL. Between-group comparisons: Mann–Whitney *U*-test, Pearson’s 𝜒2 test, statistical significance *p* < 0.05 (bold).

Based on the preliminary data analysis, which showed a high agreement between children’s attachment to the mother and attachment to the father (95% of children showed the same two-way classification for both parents), only attachment to the mother (*N* = 205) was used in further analysis to avoid multicollinearity between the independent variables. Thus, two cases were excluded because we only had information on attachment to the father. There was also a moderate association between parents’ attachment anxiety and parents’ attachment avoidance (*r* (91) = 0.43, *p* < 0.001). We only included attachment anxiety in our models for the same reason as later, to avoid multicollinearity.

### 3.2 Security of attachment and psychopathology

When comparing psychopathology, insecurely attached children had higher scores in several categories in the CBCL/YSR, but only the CBCL/YSR category withdrawn/depressed and the CBCL category social problems reached statistical significance (*p* < 0.05) (Table 5). In the 4-way classification, children classified as disorganized had higher scores in all domains of the CBCL/YSR, many of which reached statistical significance: CBCL/YSR withdrawn/depressed (*p* < 0.05), CBCL/YSR social problems (*p* < 0.01), CBCL/YSR internalizing problems (*p* < 0.05; *p* < 0.01), CBCL/YSR total score (*p* < 0.05; *p* < 0.01), CBCL somatic complaints (*p* < 0.05), CBCL aggressive behavior (*p* < 0.05), YSR anxious/depressive (*p* < 0.01), and YSR thought problems (*p* < 0.01) (Table 5). The agreement between parents and adolescents (CBCL and YSR for the same scales) measured by the Pearson correlation coefficient was statistically significant in all problem scales, ranging from 0.24 to 0.43 (*p* < 0.01).

**Table 5:** Comparison of psychopathology between securely and insecurely attached children/adolescents.

Psychopathology on ASEBA questionnaire	CAI 2-way classification		*p*	Psychopathology on ASEBA questionnaire	CAI 4-way classification		*p*
	Securely attached children	Insecurely attached children			Secure/dismissing/preoccupied	Disorganized	
	*N* = 113	*N* = 79			*N* = 162	*N* = 30	
CBCL anxious/depressed	2.55 ± 2.53 (2.00)	2.58 ± 2.50 (2.00)	0.907	CBCL anxious/depressed	2.44 ± 2.37 (2.00)	3.20 ± 3.12 (2.00)	0.349
CBCL withdrawn/depressed	1.19 ± 1.47 (1.00)	1.96 ± 2.15 (1.00)	**0.014**	CBCL withdrawn/depressed	1.38 ± 1.75 (1.00)	2.20 ± 2.02 (2.00)	**0.024**
CBCL somatic complaints	1.29 ± 1.74 (1.00)	1.43 ± 1.69 (1.00)	0.616	CBCL somatic complaints	1.19 ± 1.49 (1.00)	2.17 ± 2.49 (1.50)	**0.042**
CBCL social problems	1.09 ± 1.53 (1.00)	1.94 ± 2.30 (1.00)	**0.026**	CBCL social problems	1.22 ± 1.70 (1.00)	2.60 ± 2.60 (2.00)	**0.003**
CBCL thought problems	1.38 ± 1.73 (1.00)	1.76 ± 1.92 (1.00)	0.153	CBCL thought problems	1.46 ± 1.77 (1.00)	1.93 ± 2.02 (1.50)	0.178
CBCL attention problems	2.69 ± 3.08 (1.00)	3.24 ± 3.33 (2.00)	0.235	CBCL attention problems	2.73 ± 3.11 (2.00)	3.90 ± 3.47 (4.00)	0.088
CBCL rule-breaking behavior	1.19 ± 1.50 (1.00)	1.62 ± 1.88 (1.00)	0.250	CBCL rule-breaking behavior	1.28 ± 1.62 (1.00)	1.83 ± 1.90 (2.00)	0.138
CBCL aggressive behavior	3.04 ± 3.23 (2.00)	3.68 ± 3.85 (2.00)	0.391	CBCL aggressive behavior	3.00 ± 3.23 (2.00)	4.90 ± 4.43 (4.00)	**0.032**
CBCL internalizing problems	4.89 ± 4.24 (4.00)	5.97 ± 4.94 (5.00)	0.147	CBCL internalizing problems	4.99 ± 4.18 (4.00)	7.23 ± 5.93 (6.00)	**0.048**
CBCL externalizing problems	4.32 ± 4.43 (3.00)	5.30 ± 5.28 (4.00)	0.353	CBCL externalizing problems	4.35 ± 4.48 (3.00)	6.73 ± 5.98 (6.00)	0.061
CBCL total psychopathology	16.04 ± 12.56 (14.00)	20.19 ± 15.75 (15.00)	0.105	CBCL total psychopathology	16.49 ± 12.92 (14.00)	24.50 ± 17.94 (25.50)	**0.026**
	*N* = 71	*N* = 55			*N* = 109	*N* = 17	
YSR anxious/depressed	5.03 ± 3.99 (4.00)	5.10 ± 4.38 (4.00)	0.784	YSR anxious/depressed	4.60 ± 3.92 (4.00)	7.94 ± 4.51 (7.00)	**0.004**
YSR withdrawn/depressed	2.72 ± 2.47 (2.00)	3.71 ± 2.94 (3.00)	**0.040**	YSR withdrawn/depressed	2.86 ± 2.47 (2.00)	5.00 ± 3.54 (4.00)	**0.011**
YSR somatic complaints	3.11 ± 2.82 (2.00)	2.55 ± 2.18 (2.00)	0.333	YSR somatic complaints	2.84 ± 2.64 (2.00)	3.00 ± 2.09 (3.00)	0.537
YSR social problems	2.85 ± 2.75 (2.00)	2.73 ± 2.26 (3.00)	0.850	YSR social problems	2.54 ± 2.52 (2.00)	4.41 ± 2.06 (4.00)	**0.001**
YSR thought problems	3.76 ± 2.88 (3.00)	4.15 ± 3.59 (4.00)	0.787	YSR thought problems	3.60 ± 2.97 (3.00)	6.06 ± 3.85 (6.00)	**0.008**
YSR attention problems	4.59 ± 3.07 (4.00)	4.95 ± 3.49 (4.00)	0.633	YSR attention problems	4.45 ± 2.97 (4.00)	6.65 ± 4.30 (5.00)	0.056
YSR rule-breaking behavior	2.49 ± 1.97 (2.00)	2.84 ± 2.36 (2.00)	0.543	YSR rule-breaking behavior	2.53 ± 2.05 (2.00)	3.35 ± 2.62 (3.00)	0.238
YSR aggressive behavior	5.08 ± 3.35 (5.00)	5.35 ± 3.88 (5.00)	0.855	YSR aggressive behavior	4.94 ± 3.36 (5.00)	6.88 ± 4.53 (7.00)	0.102
YSR internalizing problems	10.86 ± 7.75 (9.00)	11.33 ± 7.85 (10.00)	0.640	YSR internalizing problems	10.30 ± 7.59 (9.00)	15.94 ± 7.33 (17.00)	**0.003**
YSR externalizing problems	7.58 ± 4.72 (6.00)	8.18 ± 5.52 (7.00)	0.655	YSR externalizing problems	7.47 ± 4.79 (6.00)	10.24 ± 6.29 (10.00)	0.098
YSR-total psychopathology	32.13 ± 18.33 (28.00)	34.44 ± 21.36 (31.00)	0.638	YSR-total psychopathology	30.83 ± 18.61 (27.00)	47.88 ± 20.36 (44.00)	**0.003**

Data are mean ± standard deviation (median). CBCL = child behavior checklist; YSR = youth self-report. Between-group comparisons: Mann–Whitney *U*-test. Bold values indicates statically significant.

### 3.3 Psychopathology and sex

A comparison of psychopathology between the sexes showed that boys scored higher than girls in all CBCL domains, apart from somatic complaints. The difference was statistically significant for thinking problems (*p* < 0.011), attention problems (*p* < 0.01) and the total score for psychopathology (*p* < 0.05). Although girls tended to report more internalizing and boys more externalizing and attention problems, the YSR sex comparison only showed a statistically significant difference for somatic complaints, where girls had higher scores (*p* < 0.05) (Table 6).

**Table 6:** Comparison of psychopathology between sexes.

CBCL scales	Female	Male	*p*	YSR scales	Female	Male	*p*
	*N* = 102/103*	*N* = 91			*N* = 72	*N* = 56	
CBCL anxious/depressed	2.53 ± 2.33 (2.00)	2.59 ± 2.70 (2.00)	0.974	YSR anxious/depressed	5.78 ± 4.66 (5.00)	4.18 ± 3.39 (3.50)	0.076
CBCL withdrawn/depressed	1.27 ± 1.63 (1.00)	1.80 ± 1.99 (1.00)	0.051	YSR withdrawn/depressed	3.24 ± 2.95 (2.50)	3.07 ± 2.43 (3.00)	0.938
CBCL somatic complaints	1.49 ± 1.94 (1.00)	1.20 ± 1.41 (1.00)	0.473	YSR somatic complaints	3.18 ± 2.45 (3.00)	2.43 ± 2.65 (2.00)	**0.041**
CBCL social problems	1.21 ± 1.78 (1.00)	1.74 ± 2.11 (1.00)	0.067	YSR social problems	3.03 ± 2.79 (2.50)	2.53 ± 2.26 (2.00)	0.466
CBCL thought problems	1.18 ± 1.39 (1.00)	1.92 ± 2.13 (1.00)	**0.011**	YSR thought problems	4.22 ± 3.31 (4.00)	3.50 ± 3.01 (3.00)	0.225
CBCL attention problems	2.21 ± 2.73 (1.00)	3.70 ± 3.47 (3.00)	**0.001**	YSR attention problems	4.46 ± 3.31 (4.00)	5.07 ± 3.19 (4.00)	0.226
CBCL rule-breaking behavior	1.29 ± 1.70 (1.00)	1.54 ± 1.86 (1.00)	0.477	YSR rule-breaking behavior	2.64 ± 2.42 (2.00)	2.80 ± 2.11 (2.00)	0.425
CBCL aggressive behavior	3.05 ± 3.62 (2.00)	3.71 ± 3.53 (3.00)	0.075	YSR aggressive behavior	5.00 ± 3.55 (5.00)	5.50 ± 3.78 (5.00)	0.485
CBCL internalizing problems	5.15 ± 4.46 (4.00)	5.59 ± 4.68 (5.00)	0.444	YSR internalizing problems	12.19 ± 8.41 (11.00)	9.68 ± 6.89 (8.00)	0.090
CBCL externalizing problems	4.44 ± 5.05 (3.00)	5.25 ± 4.94 (4.00)	0.136	YSR externalizing problems	7.64 ± 5.32 (7.00)	8.30 ± 5.26 (7.00)	0.420
CBCL total psychopathology	15.93 ± 13.72 (12.00)	20.12 ± 14.68 (18.00)	**0.021**	YSR total psychopathology	34.96 ± 20.86 (31.50)	31.12 ± 18.97 (26.00)	0.299

Data are mean ± standard deviation (median). CBCL = child behavior checklist; YSR = youth self-report. Between-group comparisons: Mann–Whitney *U*-test. *In category CBCL somatic complaints we only had 102 answers (*N* = 102). Bold values indicates statically significant.

### 3.4 Psychopathology and attachment insecurity of parents

There were significant correlations between the child’s psychopathology and the parents’ attachment insecurity (anxiety (r = 0.18 to 0.23) and avoidance (*r* = 0.14 to 0.28)). Parental attachment anxiety was statistically significantly related to the anxious/depressed scale and internalizing problems on both the CBCL (*r* = 0.21–0.22; *p* < 0.01) and YSR (*r* = 0.18–0.21; *p* < 0.05) scales, social problems and the total psychopathology score on the CBCL scale (*r* = 0.20–0.21; *p* < 0.01). Parental attachment avoidance was statistically significantly related to anxious/depressed, withdrawn/depressed, social problems, internalizing problems, and total score on both the CBCL and YSR scales (*r* = 0.16–0.304; *p* < 0.01 or *p* < 0.05) and with somatic complaints, thinking problems and rule-breaking behavior on the YSR scale (*r* = 0.24–0.28; *p* < 0.01 or *p* < 0.05) (Table 7).

**Table 7:** Correlation between psychopathology and attachment security of parents.

CBCL scales	ECR-RS-anxiety		YSR scales	ECR-RS-anxiety		CBCL scales	ECR-RS-avoidance		YSR scales	ECR-RS-avoidance	
	*N* = 190			*N* = 121			*N* = 190			*N* = 121	
	*r*	*p*		*r*	*p*		*r*	*p*		*r*	*p*
CBCL anxious/depressed	0.22	**0.002**	YSR anxious/depressed	0.21	**0.019**	CBCL anxious/depressed	0.23	**0.001**	YSR anxious/depressed	0.24	**0.007**
CBCL withdrawn/depressed	0.09	0.236	YSR withdrawn/depressed	0.05	0.554	CBCL withdrawn/depressed	0.19	**0.010**	YSR withdrawn/depressed	0.28	**0.002**
CBCL somatic complaints	0.14	0.051	YSR somatic complaints	0.16	0.086	CBCL somatic complaints	0.12	0.097	YSR somatic complaints	0.24	**0.008**
CBCL social problems	0.23	**0.001**	YSR social problems	0.12	0.194	CBCL social problems	0.21	**0.004**	YSR social problems	0.28	**0.002**
CBCL thought problems	0.12	0.110	YSR thought problems	0.18	0.051	CBCL thought problems	0.04	0.614	YSR thought problems	0.19	**0.036**
CBCL attention problems	0.04	0.611	YSR attention problems	−0.03	0.735	CBCL attention problems	0.02	0.772	YSR attention problems	0.07	0.467
CBCL rule-breaking behavior	0.13	0.067	YSR rule-breaking behavior	0.09	0.335	CBCL rule-breaking behavior	0.16	0.028	YSR rule-breaking behavior	0.24	**0.008**
CBCL aggressive behavior	0.14	0.062	YSR aggressive behavior	0.02	0.803	CBCL aggressive behavior	0.06	0.374	YSR aggressive behavior	0.05	0.574
CBCL internalizing problems	0.21	**0.003**	YSR internalizing problems	0.18	**0.043**	CBCL internalizing problems	0.24	**0.001**	YSR internalizing problems	0.30	**0.001**
CBCL externalizing problems	0.14	0.051	YSR externalizing problems	0.05	0.556	CBCL externalizing problems	0.12	0.106	YSR externalizing problems	0.14	0.128
CBCL total psychopathology	0.20	**0.007**	YSR total psychopathology	0.13	0.150	CBCL total psychopathology	0.16	**0.024**	YSR total psychopathology	0.25	**0.006**

ECR-RS-anxiety = ECR-RS relationship structures questionnaire–common score for anxiety in mothers; CBCL = child behavior checklist, YSR = youth self-report; *r* = Pearson’s correlation coefficient. Bold values indicates statically significant.

### 3.5 Psychopathology and traumatic life events

Parents’ reports of traumatic life events were statistically significantly related to psychopathology as rated by parents, but not by children. Children’s ratings of stressful life events, however, were associated with some psychopathology scales (total psychopathology score, as well as anxious/depressed, thought problems, rule-breaking behavior, and the internalizing scale) (Table 8).

**Table 8:** Correlation between psychopathology and traumatic life events.

CBCL scales	LITE-P		LITE-Y		YSR scales	LITE-P		LITE-Y	
	*N* = 192		*N* = 194			*N* = 122		*N* = 128	
	*r*	*p*	*r*	*p*		*r*	*p*	*r*	*p*
CBCL anxious/depressed	0.22	**0.002**	0.27	**0.000**	YSR anxious/depressed	−0.09	0.343	0.27	**0.005**
CBCL withdrawn/depressed	0.18	**0.011**	0.15	**0.034**	YSR withdrawn/depressed	−0.03	0.698	0.12	0.164
CBCL somatic complaints	0.18	**0.014**	0.16	**0.03**	YSR somatic complaints	−0.00	0.985	0.04	0.660
CBCL social problems	0.31	**0.000**	0.28	**0.000**	YSR social problems	0.06	0.489	0.17	0.061
CBCL thought problems	0.29	**0.000**	0.22	**0.002**	YSR thought problems	0.04	0.631	0.22	**0.012**
CBCL attention problems	0.20	**0.006**	0.28	**0.000**	YSR attention problems	0.00	0.989	0.17	0.057
CBCL rule-breaking behavior	0.11	0.127	0.18	**0.012**	YSR rule-breaking behavior	−0.12	0.174	0.23	**0.008**
CBCL aggressive behavior	0.19	**0.008**	0.31	**0.000**	YSR aggressive behavior	−0.01	0.903	0.03	0.765
CBCL internalizing problems	0.26	**0.000**	0.26	**0.000**	YSR internalizing problems	−0.06	0.514	0.19	**0.033**
CBCL externalizing problems	0.20	**0.005**	0.30	**0.000**	YSR externalizing problems	−0.06	0.504	0.12	0.180
CBCL total psychopathology	0.29	**0.000**	0.32	**0.000**	YSR total psychopathology	−0.06	0.499	0.20	**0.021**

LITE-P = lifetime incidence of traumatic events–parents’ version; LITE-Y = lifetime incidence of traumatic events–youth’ version; CBCL = child behavior checklist; YSR = youth self-report; *r* = Pearson’s correlation coefficient. Bold values indicates statically significant.

### 3.6 Linear regression models analysis

Due to the higher number of complete cases and to avoid multicollinearity between parent- and child-reported measures, only parent-reported psychopathology (CBCL) and adverse life events (LITE-P) were included in the subsequent analyses. The results of the multivariate linear regression models showed that the significant predictors of psychopathology were attachment insecurity, especially attachment disorganization, higher parental attachment anxiety and traumatic life events. T1D was not a statistically significant predictor in this model (Table 9).

**Table 9:** Multivariate linear regression model indicating predictors of general psychopathology (CBCL), model without interactions between the independent variables.

CBCL							
Model 1	*R* ^2^	Adj. *R*^2^	*p*	Model 2	*R* ^2^	Adj. *R*^2^	*p*
	15.9%	14.1%	<0.001		16.7%	14.9%	<0.001

	*B*	*t*	*p*		*B*	*t*	*p*
LITE-P	2.595	4.632	**<0.001**	LITE-P	2.470	4.416	**<0.001**
ECR-RS-anx	2.838	2.400	**0.017**	ECR-RS-anx	2.386	1.990	**0.048**
CAI Sec/Insec	−4.082	−2.091	**0.038**	Disorganization	6.577	2.465	**0.015**
T1D	−2.615	−1.339	0.182	T1D	−2.935	−1.499	0.136

ECR-RS-anx: ECR-RS relationship structures questionnaire–common score for anxiety in mothers; LITE-P: lifetime incidence of traumatic events parents’ version; CAI Sec/Insec: child attachment interview 2-way classification: 1 = secure, 0 = insecure; Disorganization: disorganized attachment in 4-way classification; T1D: type 1 diabetes; Adj. *R*^2^ = adjusted *R*^2^. Bold values indicates statically significant.

In the subsequent analyses, we constructed multivariate linear regression models including sex, age, and selected interaction terms, guided by a biopsychosocial framework in which psychopathology is understood to arise from interactions between biological and psychosocial factors across development (Cicchetti and Rogosch, 2002).

**Figure 1:**
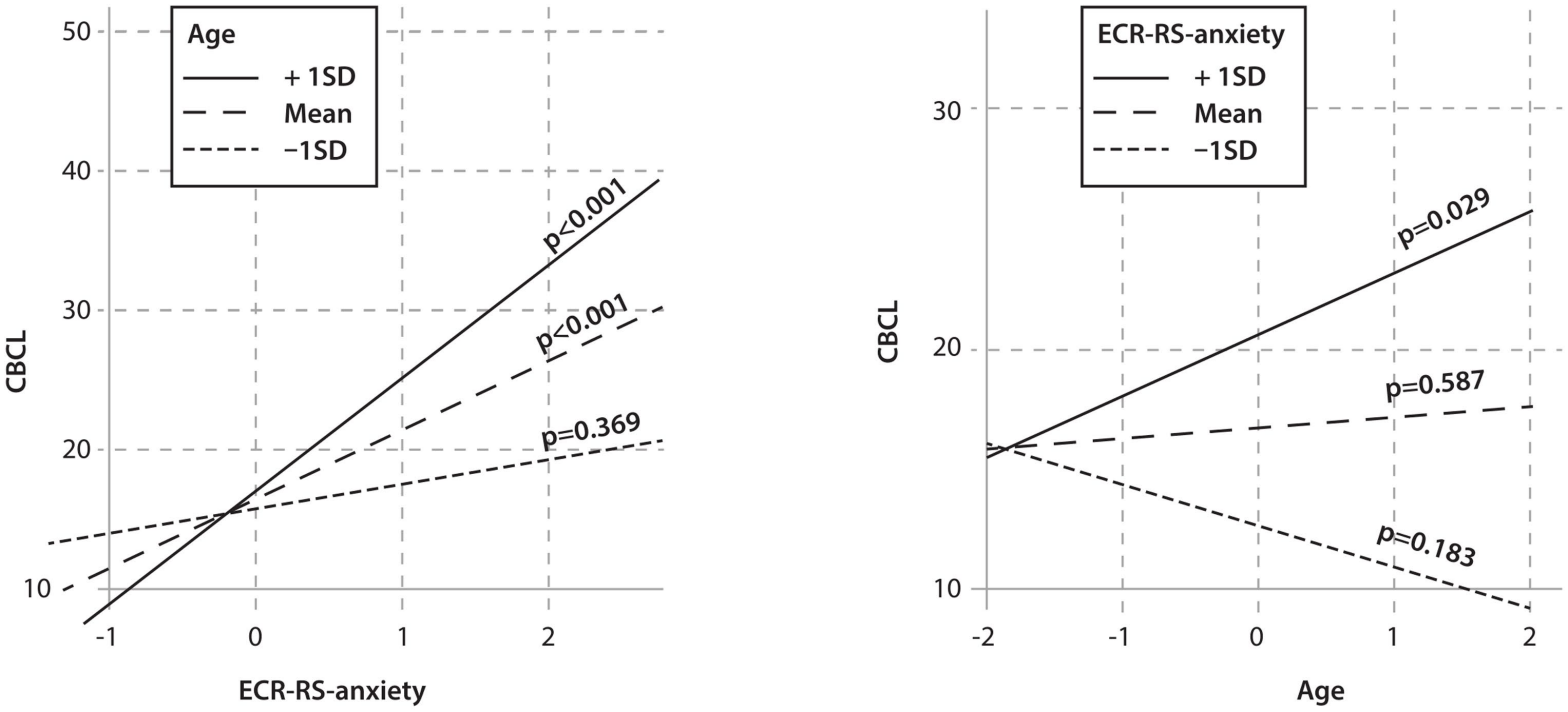
Linear regression model—the relationship between parental attachment anxiety, child age, and psychopathology in children. CBCL: Child Behavior Checklist; ECR-RS-anxiety: ECR-RS relationship structures questionnaire–common score for anxiety in mothers; Age: age in years.

**Figure 2:**
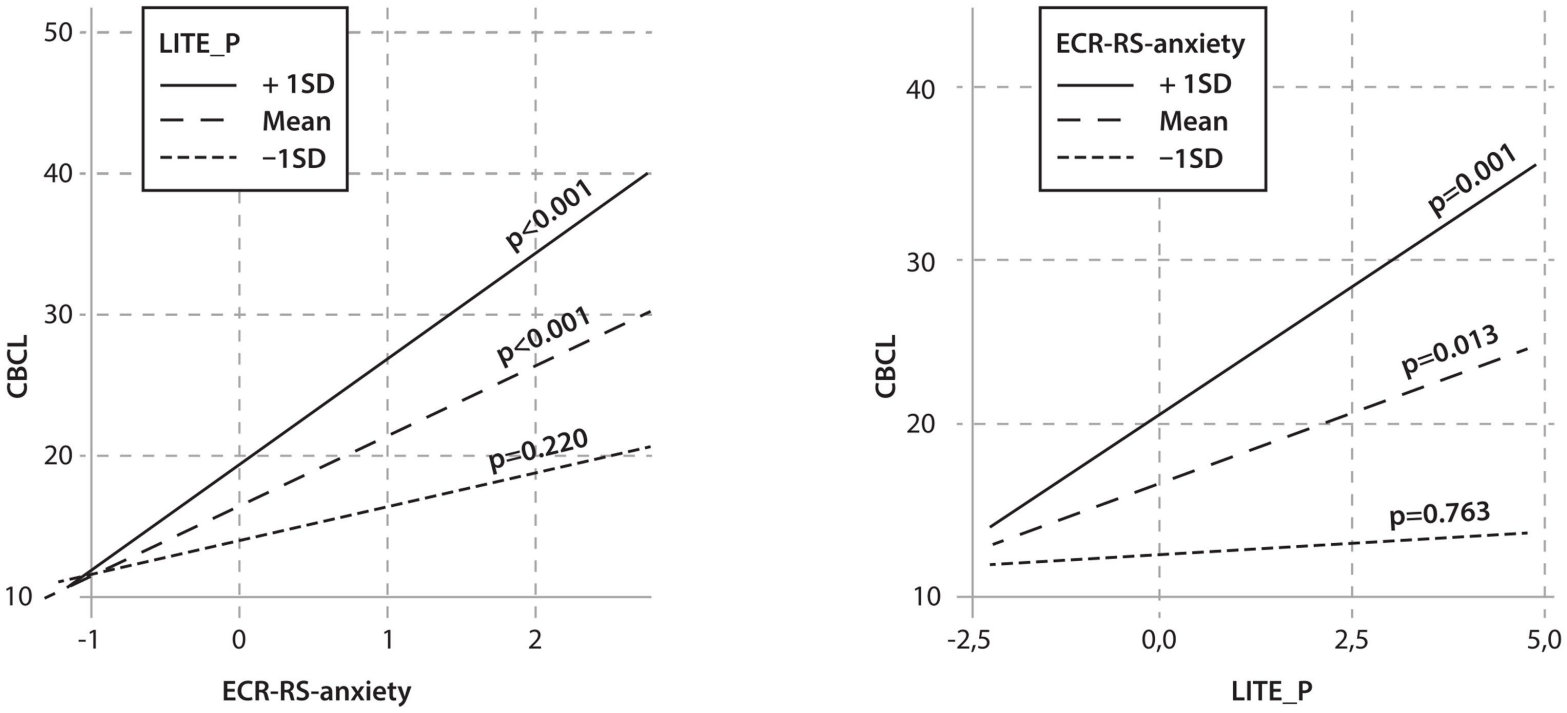
Linear regression model—the relationship between parents’ attachment anxiety, children’s traumatic events, and children’s psychopathology. CBCL: Child Behavior Checklist; ECR-RS-anxiety: ECR-RS relationship structures questionnaire–common score for anxiety in mothers; LITE_P: Lifetime incidence of traumatic events (parents’ version).

The results showed that the child’s male sex and the presence of T1D were significant predictors of psychopathology. Two interactions between independent variables showed statistical significance, namely the interaction between parental attachment anxiety and age and between parental attachment anxiety and traumatic life events (Table 10). Simple slope analyses were calculated for these interactions, which are shown in Figures 1, 2.

**Table 10:** Multivariate linear regression model indicating predictors of general psychopathology, model with interactions between independent variables.

CBCL	*R* ^2^	Adj. *R*^2^	*p*
	33.03%	*27.4%*	<0.001

	*B*	*t*	*p*
Sex	−6.24	−2.91	**0.004**
Age	0.46	0.54	0.59
ECR-RS-anx	4.90	3.80	**<0.001**
LITE-P	1.67	2.52	**0.013**
CAI Secure/insecure	0.85	0.29	0.77
T1D	8.83	2.58	**0.011**
Age x ECR-RS anxiety	2.59	2.44	**0.016**
ECR-RS-anxiety x LITE-P	1.69	2.24	**0.027**
CAI Sec/Insec x T1D	−7.72	−1.76	0.081

Sex: 1 = female; 0 = male; Age: age of a child in years; ECR-RS-anx: ECR-RS relationship structures questionnaire–common score for anxiety in mothers; LITE-P: lifetime incidence of traumatic events (parents’ version); CAI security: child attachment interview 2-way classification; T1D: type 1 diabetes. Bold values indicates statically significant.

The analysis of interactions showed significant correlations between parental attachment anxiety and psychopathology as a function of age. Middle-aged and older children whose mothers showed more attachment anxiety had higher psychopathology scores. When parents reported high levels of attachment anxiety, there was also a significant relationship between age and psychopathology (Figure 1).

The interaction analysis also showed that children with an average and higher number of traumatic life events whose mothers had higher attachment anxiety had higher psychopathology scores. When parents had average or higher levels of attachment anxiety, there was also a significant relationship between the number of traumatic life events experienced by the child and the child’s psychopathology (Figure 2).

## 4 Discussion

The present study aimed to examine whether attachment quality and related factors were associated with psychopathology in children with T1D compared to healthy peers. Children with T1D did not differ from healthy children in overall levels of psychopathology. Across groups, however, insecure attachment—particularly disorganized attachment—was associated with higher psychopathology scores. These findings suggest that attachment-related vulnerabilities are relevant for psychological adjustment in children regardless of T1D status. Further analyses indicated that developmental and contextual variables, including child age, sex, parental attachment anxiety, and exposure to traumatic life events, contributed to psychopathology, highlighting the importance of considering multiple interacting influences when examining mental health outcomes in children with T1D.

### 4.1 Security of attachment and psychopathology

In our cohort, 59% of the children were classified as securely attached. Previous studies using the same measures have reported a slightly higher proportion of securely attached children (66%) (Costa-Cordella et al., 2020). Among the insecurely attached children, the majority were dismissive (39%), which is also slightly higher than in the study by Shmueli-Goetz et al. (2008) (30%), 2% were classified as preoccupied, which is lower than in the previously mentioned study (4%). In our sample, 16% of children were disorganized in the 4-way classification, which is much higher compared to the 4% in the study by Shmueli-Goetz et al. (2008).

Our results demonstrate a significant association between disorganized attachment and multiple domains of psychopathology in both groups, given that no differences in attachment representations were observed between children with T1D and healthy controls (Table 1). Previous studies have identified insecure attachment as a risk factor for internalizing and externalizing psychopathology in childhood and adolescence (Fearon et al., 2010; Groh and Narayan, 2019). Within insecure attachment, a disorganized attachment style between child and parent has been associated with neurological vulnerability in the child and thus with a range of later social and cognitive difficulties as well as psychopathology (van Ijzendoorn et al., 1999). Disorganized attachment is related to an incoherent internal representation of the same parent who simultaneously evokes the need for closeness and anxiety (Green and Goldwyn, 2002). This results in difficulties in stress and emotion regulation, impaired mentalization (the ability to understand mental states of self and others), and interpersonal dysfunction, which together increase the risk of psychopathology (Luyten et al., 2020).

Our findings suggest that, when controlling for other variables, child attachment security may play a lesser role than factors such as parental attachment security, traumatic life events, age, sex, and the presence of a chronic illness like T1D. Parental attachment insecurity appears to be a more important risk factor than child insecurity, which may indicate that the parents’ emotional well-being and their ability to regulate the child’s stress are more important than the intrinsic abilities of the child itself (Nelson et al., 2009).

However, the population included in our study was non-clinical in terms of psychopathology. It is possible that in a clinical psychiatric sample of adolescents, the influence of attachment on psychopathology would be stronger. This can be inferred from a study of psychiatric inpatients that included adolescents with psychiatric disorders such as depression, obsessive-compulsive disorder, and oppositional defiant disorder which showed an inverse distribution of attachment security (30.4% secure and 69.5% insecure attachment) (Venta et al., 2014).

### 4.2 T1D and psychopathology

There were no differences in psychopathology when comparing children with and without T1D. However, findings in the literature remain inconsistent. Some studies have reported a higher incidence of mental disorders, such as depression, anxiety and eating disorders, among children with T1D, while others have found a lower or similar incidence compared with healthy children (Sivertsen et al., 2014; Dybdal et al., 2018). These discrepancies may be partly explained by differences in age range, methods used to assess psychopathology, and variations in access to specialized psychosocial care.

Children and adolescents at different developmental stages face distinct challenges in both mental health and diabetes management (Cicchetti and Rogosch, 2002; Berg et al., 2017). Younger children typically depend on their parents for daily diabetes care and may show fewer overt signs of depression or anxiety (Berg et al., 2017). In contrast, adolescents enter a critical period of biological, emotional, and social change, during which they increasingly assume responsibility for self-management (Bombaci et al., 2024). Puberty, hormonal fluctuations, and psychosocial stressors can impair metabolic control and increase disease-related stress, making this a particularly vulnerable period for psychological difficulties (Bombaci et al., 2024). As further discussed, our results highlight the importance of age and sex differences in the relationship between T1D and psychopathology.

Some studies focused on subclinical symptoms, while others assessed formal psychiatric diagnoses, which may have further contributed to divergent results (Dybdal et al., 2018; Telo et al., 2018). One large population-based sample showed that mental health symptoms were more common in adolescents with T1D than in healthy controls, whereas diagnoses of common mental disorders were not more prevalent, suggesting increased subclinical psychological distress rather than overt psychopathology (Telo et al., 2018). A Slovenian study involving 126 adolescents with T1D and 499 healthy adolescents showed that the prevalence of suicidal thoughts, suicide attempts and self-harming behavior was lower among the adolescents with T1D than among healthy peers (Drobnič Radobuljac et al., 2009). At the same time, the prevalence of eating disorders was higher among female adolescents with T1D (Drobnič Radobuljac et al., 2013). In this cohort, most adolescents were treated with insulin pumps, reflecting well-organized, multidisciplinary diabetes care, which may contribute to favourable mental health outcomes (Turin et al., 2021). Nevertheless, within the same T1D cohort, suicidal behavior was associated with poorer metabolic control (Drobnič Radobuljac et al., 2009), indicating that vulnerable subgroups remain at increased risk. Consistently, larger population-based studies have demonstrated an association between poor metabolic outcomes and psychiatric comorbidities (Sildorf et al., 2018).

Our results showed that the presence of T1D has no significant effect on psychopathology when the individual variables were analyzed. In contrast, when interactions among variables such as parental attachment anxiety, child age, and traumatic life events were considered, T1D was associated with an increased risk of psychopathology. Male sex and T1D were associated with higher CBCL total scores (main effects). Consistent with the earlier discussion on developmental stage, age moderated the association between general anxiety and CBCL scores: the relationship was significant at the mean age and +1 SD, but not at −1 SD, highlighting adolescence as a period of increased vulnerability to psychological difficulties in the context of T1D (Bombaci et al., 2024).

The association between maternal attachment anxiety was also statistically significant in children with average or higher frequency of negative life experiences reported by parents. According to the results, special attention should be paid to boys with T1D who have been exposed to more negative life events and whose mothers appear to be less securely attached. Since the association between attachment anxiety and psychopathology was only observed in middle-aged (Mean age 11.7 years) or older children, special attention should be paid to adolescent boys compared to younger children.

A previous study on the same cohort of children also highlighted the sex differences in metabolic outcomes of T1D. The results showed that in boys there was a positive correlation between parental attachment style and glycemic outcomes. The more insecure the parental attachment, the higher the mean Hb1Ac, HbA1c variability and the lower the TIR (time in required glycemic range). The reverse relationship was observed in girls (Turin and Drobnič, 2021). This may be explained by the transfer of responsibility for disease management from parents to adolescents, with more insecurely attached parents possibly being more anxious and controlling, which may be beneficial in younger children but may hinder autonomous motivation and information sharing in adolescents (Herzer and Hood, 2010). A trusting relationship has been shown to be a protective factor for good glycemic control in adolescence (Berg et al., 2017). In securely attached girls, the transfer of responsibility for T1D management may have occurred earlier, possibly exceeding their maturity and leading to suboptimal metabolic outcomes (Klemencic et al., 2023).

### 4.3 Strengths and limitations

A strength of the study is the recruitment of nearly the entire cohort of Slovenian children with T1D aged 8–15 years, using a validated qualitative interview analyzed by three independent coders to assess attachment. This is the first Slovenian survey that allows comparisons of attachment profiles with international studies.

A limitation of the study is a rather homogeneous nature of the cohort, as all children identified as white and most used insulin pumps, limiting generalizability. We used a qualitative approach to assess children’s attachment to their parents, but parents’ attachment was only assessed using a self-report questionnaire, which, despite its reliability and validity, is at risk of over- or under-reporting. Our cohort was not clinical in terms of psychopathology, and we only had information on symptoms based on self/parent evaluations. Our results also showed an association between mothers’ attachment anxiety and parent-rated psychopathology, suggesting that lower maternal stress regulation influences children’s psychopathology, but may also indicate that mothers with higher attachment anxiety exaggerate their children’s symptoms. The exclusive use of parent ratings was aimed at increasing the power of the study by obtaining more CBCL reports.

## 5 Conclusion

In this study, we examined the role of children’s attachment to their parents and parental attachment security in relation to child psychopathology, focusing on children with T1D. Our results indicate that older age, male sex, the presence of T1D, parental attachment insecurity, and greater exposure to traumatic life events were all associated with higher parent-rated child psychopathology. These contextual and parental factors appeared stronger predictors than the child’s own attachment security. Nevertheless, disorganized attachment in the child was strongly associated with multiple domains of psychopathology, consistent with previous research on attachment and child mental health.

These findings highlight the importance of considering multiple, interacting factors when addressing psychological adjustment in children and adolescents with T1D. Clinical care and prevention efforts should account for developmental and sex-specific needs, particularly during the transition of disease management from parents to adolescents. Furthermore, integrating evidence-based attachment and trauma-informed approaches into routine practice may enhance both the psychological well-being of children and parents and the management of T1D.

## Data Availability

The data are available from the corresponding author upon reasonable request and subject to ethical approval.

## Footnotes

1
www.ASEBA.org
